# The Efficacy of Processing Strategies on the Gastroprotective Potentiality of *Chenopodium quinoa* Seeds

**DOI:** 10.1155/2020/6326452

**Published:** 2020-05-28

**Authors:** Abdalbasit Adam Mariod, Suzy Munir Salama

**Affiliations:** ^1^College of Sciences and Arts-Alkamil, University of Jeddah, Alkamil, Saudi Arabia; ^2^Department of Biomedical Science, Faculty of Medicine, University of Malaya, 50603 Kuala Lumpur, Malaysia

## Abstract

The current study has been conducted to evaluate the effect of different processing techniques on the 2,2-diphenyl-1-picrylhydrazyl (DPPH) scavenging capacity and the gastroprotective potential of *Chenopodium quinoa* red seeds in acute gastric injury induced by absolute ethanol in rats. Seven groups of female Sprague Dawley rats were assigned to normal and absolute ethanol (absolute EtOH) groups, given distilled water, reference control omeprazole (OMP, 20 mg/kg), pressure-cooked quinoa seeds (QP, 200 mg/kg), first stage-germinated quinoa seeds (QG, 200 mg/kg), *Lactobacillus plantarum* bacteria-fermented quinoa seeds (QB, 200 mg/kg), and *Rhizopus oligosporus* fungus-fermented quinoa seeds (QF, 200 mg/kg). One hour after treatment, all groups were given absolute ethanol, except for the normal control rats. All animals were sacrificed after an additional hour, and the stomach tissues were examined for histopathology of hematoxylin and eosin staining, immunohistochemistry of cyclooxygenase 2 (COX-2), and nitric oxide synthase (iNOS). Stomach homogenates were evaluated for oxidative stress parameters and prostaglandin E_2_ (PGE_2_). Gene expression was performed for gastric tumor necrosis factor alpha (TNF-*α*) and nuclear factor kappa of B cells (NF-kB). QB and QG recorded the highest DPPH scavengers compared to QF and QP. The gastroprotective potential of QB was comparable to that of OMP, followed by QF, then QG, and QP as confirmed by the histopathology, immunohistochemistry, and gene expression assessments. In conclusion, differently processed red quinoa seeds revealed variable antioxidant capacity and gastroprotective potential, while the bacterial fermented seeds (QB) showed the highest potential compared to the other processing techniques. These results might offer promising new therapy in the treatment of acute gastric injury.

## 1. Introduction

The genus *Chenopodium* has attracted the studies of researchers interested in the therapeutic potential of its different species such as the antibacterial effect [1] and anti-inflammatory activity [2] of *Chenopodium ambrosioides*. Another study on *Chenopodium album* revealed antioxidant activity of phenolic glycoside isolated from its seeds [3]. *Chenopodium quinoa* Willd. is a *Chenopodium* species that is indigenous to the Andean sector [4]. Recently, quinoa seeds are consumed worldwide mainly similarly to wheat, rice, and corn and barley for their distinctive, high nutritive, and healthy values [5]. According to Food and Agriculture Organization (FAO), the seeds of quinoa comprise favorable-grade B vitamins compared to other consumable grains [6]. Additionally, quinoa seeds are considered as richer source of minerals (zinc, magnesium, calcium, and iron) than other conventional cereals [7]. Moreover, quinoa seeds have anthocyanins and polyphenols which are highly active antioxidants [8]. On the nonnutritional side, the seed taste of quinoa contains bitter substances called saponins which are water-soluble and toxic but can be removed easily by washing before cooking [4].

At the pathology level, the characteristics of gastric lesions in rats are similar to those in humans [9]. Therefore, rat models have been used for decades by many researchers' *in vivo* studies to evaluate the gastroprotective potential of drugs [10–12]. Due to the global demands of quinoa seeds, researchers paid more attention to exploring more about the chemical composition and the benefits of these seeds. Gluten-sensitive people consider quinoa seeds as the most convenient cereal due to their gluten-free nature [13]. Additionally, the chemical profile of quinoa seeds revealed different phytoconstituents including alkaloids such as betanin [14], flavonols and their glycosides such as kaempferol and quercetin glycosides [15], sterols such as ecdysteroids [16], saponins [17], and terpenoids [1]. Takao et al. stated that the portion of protein separated from quinoa seeds exhibited hypocholesterolemic potential in mice [18]. The colored varieties of *Chenopodium quinoa* seeds showed significant antioxidant activity of their flavonoids and phenolic acid constituents [19]. Miranda et al. found that quinoa seeds collected from different geographical regions manifested considerable antimicrobial activities [20]. The saponins separated from the seed coats of quinoa seeds were proved to show immunomodulatory [21] and anti-inflammatory activities [5]. Recent studies found that polysaccharides isolated from quinoa seeds showed protective activity against absolute ethanol-induced acute gastric injury [22] and cytotoxic activity against human liver and breast cancer *in vitro* [23]. Another study reported that the phytoecdysteroids discharged from the grains of quinoa at the beginning of germination exerted hypoglycemic effect in experimental animals [24].

Different processing techniques were shown to determine the percentage of active ingredients released from quinoa seeds. Germination and fermentation of quinoa seeds were proved to release more effective antioxidants which are more functional against diseases [24]. Another study stated that cooking quinoa seeds in water under high heat and pressure improved the antioxidant capacity of the seeds [25]. The objective of this research is to study the impact of different processing protocols of red quinoa seeds, QP (quinoa seeds cooked under high pressure), QG (first stage-germinated quinoa seeds), QB (quinoa seeds fermented by *Lactobacillus plantarum* bacteria), and QF (quinoa seeds fermented by *Rhizopus oligosporus* fungus), on the antioxidant power and gastroprotective potential in acute gastric injury developed by absolute ethanol in rats.

## 2. Methodology

### 2.1. Preparation of Samples

High-quality red quinoa seeds (South Sinai, Egypt) packaged in January 2018 by Samo Trading Company were purchased from the health store of local market and washed thoroughly in tap water to remove toxic saponins. The washed quinoa seeds were processed differently by four different techniques as explained below.

#### 2.1.1. Cooking Seeds under High Temperature and Pressure (QP)

The cooking process was conducted following the protocol of Nickel et al. [25] with minor modification. Briefly, 100 mg of the prewashed red quinoa seeds was mixed with distilled water (1 : 3 w/v) and cooked in a pressure cooker (Seb, Ecully, France) for 6 minutes after the onset of the pressure whistle. Following cooling, the cooked seeds were blended in 100 mL distilled water using electric blender (Philips, Shanghai, China) for 5 min, and the resulting suspension was kept at −20°C till use.

#### 2.1.2. First Stage Germination of Seeds (QG)

Germination steps were done following the methodology of Carciochi et al. [26] with few changes. Prewashed red quinoa seeds were spread on wet autoclaved filter paper (Double Rings 203, 11.0 cm) inside a germination tray to reduce contamination of germinating seeds [27]. The tray was left incubated in the dark for 72 hours under controlled conditions of temperature (23 ± 2°C) and relative humidity (80%). After 4 days, the resulting sprouts were collected, air-dried for 48 hours, and then ground in an electric grinder (Philips, Shanghai, China) to fine powder. The produced powder was suspended in distilled water (1 mg/mL) and kept at 4°C one day before the experiment day.

#### 2.1.3. Fermentation of Seeds Using *Lactobacillus plantarum* Bacteria (QB)

*Lactobacillus plantarum* bacteria (ATCC 8014) was initially propagated in broth tube following ATCC instructions and incubated at 37°C for 24 hours. Fermentation procedure was performed as done in previous experiments [28]. Briefly, the broth tube was centrifuged at 10000 × g for ten minutes at 4°C to harvest bacterial cells. Sterile potassium phosphate buffer (50 mM, pH 7.2) was used to double-wash the cells which were finally resuspended in sterile distilled water to prepare them for fermentation use at density of 1.37 × 10^8^ CFU/mL. Fifty grams of the previously washed red seeds was ground in an electric grinder (Philips, Shanghai, China) to fine powder, and 10 g of the resulting powder was mixed with 30 mL of the above prepared bacterial suspension forming dough. The produced dough was left for fermentation in Lab-Therm temperature controlled shaker (Kühner, Switzerland) with stirring rate of 200 rpm for 24 hours at 37°C to release as much antioxidants as possible [28]. One gram of the fermented dough was suspended in 10 mL of distilled water and kept at −20°C till use.

#### 2.1.4. Fermentation of Seeds Using *Rhizopus oligosporus* Fungus (QF)

Fermentation protocol was conducted as per the method of Starzyńska-Janiszewska et al. [29] with slight modification. *Rhizopus oligosporus* fungus (ATCC 48010) was incubated on potato dextrose agar (PDA) at 25°C for one week till formation of black spores. The mature spores were then suspended in a mixture containing sterile saline solution at concentration of 1 g/100 mL, 0.01% Tween 80, and peptone at concentration of 0.001 g/100 mL under carefully sterilized conditions. The spore density required for fermentation (10^5^/mL) was prepared using Neubauer counting chamber (Merck, Darmstadt, Germany). Prewashed red quinoa seeds were cooked for 20 minutes in acidified tap water (1 : 3°w/v, pH = 4) and then dried on sterilized filter paper. *R. oligosporus* spores were inoculated and mixed with one gram of the dried seeds at density of 10^5^/g in sterilized and sealed Petri dish and left incubated at 35°C for germination of spores. Following spore germination, the temperature was then cooled to 30°C, and incubation continued to 48 hours to complete the fermentation process. The fermented quinoa seeds were finally blended in sterile distilled water (1 g/mL), and the resulting suspension was left at −20°C one day before use.

### 2.2. Scavenging Activity of DPPH Free Radical

The highly stabilized free radical DPPH (2,2-diphenyl-1-picrylhydrazyl) was purchased from Merck, Darmstadt, Germany, and used to evaluate the scavenging ability of QP, QG, QB, and QF. The protocol was carried out based on the method previously done using ascorbic acid (As) (Merck, Darmstadt, Germany), a well-known standard in similar tests [30, 31]. In brief, a quantity of 1 mg was measured from each type of the processed seeds/ascorbic acid and dissolved separately in 1 mL of DMSO (dimethyl sulphoxide) forming stock solution from each sample/standard. Five serial dilutions were prepared carefully from each sample to the final concentration of 3.125 *μ*g/mL. Ten microliters from each sample was set into their labeled wells of 96-well plate in triplicate, and 190 *µ*L of DPPH was added after that to each well in dark room. The reading of the spectrophotometer was taken at 517 nm, and the percentage of DPPH inhibition was estimated from the formula (OD_blank_ − OD_sample_)/(OD_blank_) × 100.

### 2.3. Animal Experiment

Forty-two female Sprague Dawley (SD) rats weighing 150–180 g were purchased from Nile Center of Experimental Research (NCER) and kept at controlled conditions of the center's animal house (24-25°C temperature and 55 ± 2% humidity) in polypropylene rat cages. The protocol was approved by the committee of NCER (15/01/2017), Mansoura, Egypt. All the animals were left acclimated for three days before the onset of the experiment and maintained at 12-hour light/dark cycle while having free access to drinking water and standard food. Following acclimation, the animals were separated into 7 labeled groups (normal, absolute EtOH, OMP, QP, QG, QB, and QF), and each group included 6 animals. All the rats were given humane care as recommended by the animal care guidelines of NCER and accredited by the International Accreditation Organization (IAO, May 07, 2014) [32]. The rats were prepared by fasting for eighteen hours before the day of the experiment with free access to water.

In the experimental design of the present study, absolute ethanol (absolute EtOH) was selected to induce gastric ulcer by oral gavage at dose of 5 mL/kg as conducted in previous protocols [33, 34]. Following 18 hours of fasting, distilled water was orally administered to normal (animals without gastric lesion and without treatment) and ulcer control group rats. Omeprazole group (OMP) was given omeprazole at dose of 20 mg/kg dissolved in distilled water and prepared one day before the experiment [35]. Experimental groups were given the different types of processed quinoa seeds (QP, QG, QB, and QF), each at dose of 200 mg/kg, and all the animals were left without access to food or water for one hour. Next, absolute EtOH was orally given to all the groups except for the normal group, while all the animals were left without food or water for an additional hour. Thereafter, all the animals were euthanized under halothane inhalation of liquid vapor (Academic International Trading, Giza, Egypt) [36, 37]. The stomachs were then excised from all rats and examined grossly for the elongated haemorrhagic lesions, while the macroscopic pictures were photographed using Canon PowerShot digital camera (Tokyo, Japan) [38]. Three tissue parts were cut from each stomach; one part was kept in normal saline for preparing stomach tissue homogenate, the second part was kept in 10% buffered formalin for histopathology and immunohistochemistry studies, and the third part (30 mg) was placed in sterile labeled cryovials and preserved in liquid nitrogen for gene expression assessment. Dose regimen of the processed seeds given to the rats was selected as 1/10 of the safe dose reported previously in rats (2000 mg/kg) [39]. Additionally, in humans the safe dose was determined as 19.5 g/day for thirty-day clinical case study [40].

#### 2.3.1. Ulcer Index Estimation

Using a dissecting microscope of magnification 1.8x equipped with eyepiece square grid (4 mm^2^/square), the total area of the haemorrhagic lesioned strips of each stomach was observed and measured in mm^2^. The % inhibition of ulceration was calculated using the following formulae and guided by previous methodologies [41],

(1) $$\begin{array}{c} \text{Total lesioned area of each stomach}=\text{sum of squares covering all the lesions}\times 4\times 1.8,\\ \text{Percentage of ulcer inhibition}=\left[\frac{\left(\text{Ulcer control area}-\text{Treated ulcer area}\right)}{\text{Ulcer control area}}\right]\times 100. \end{array}$$

#### 2.3.2. Gastric pH and Gastric Wall Mucus Assessment

The gastric acidity of the rat's stomachs was measured by pH-meter (Jenway, Stone, UK), and the mucus of the gastric wall assessed using the steps of Corne et al. [42] with few changes. Briefly, in 10 mL labeled tubes with prepared Alcian blue solution (0.1% w/v), the separated gastric mucosa from each stomach was incubated in its designated tube for 2 hours, followed by double wash with sucrose solution (0.25 M). The samples were then immersed and incubated in 10 mL of magnesium chloride solution (0.5 M) on the shaker for additional couple of hours. After incubation, diethyl ether (4 mL) was added to the samples and centrifuged at 4000 rpm for ten minutes. The color intensity of dyed samples was determined by reading the optical density at 580 nm.

#### 2.3.3. Gastric Homogenate Preparation

Separate pieces of the rat's stomachs (0.5 g) were cut and homogenized in 5 mL cold phosphate buffered saline of pH 7.3 using Tekmar Tissumizer (Cincinnati, Ohio, USA) at 4°C and 4500 rpm. The resulting supernatant from each sample was aliquoted and preserved at −20°C to be assayed within two weeks.

#### 2.3.4. Gastric Protein Content

The protein concentration in the tissue homogenate of all the rats' stomachs was determined following the procedure of Lowry et al. The assay was conducted in triplicate, and the protein concentration was interpolated from the standard curve generated using bovine serum albumen standard (BSA, 100 mg/L) after reading the absorbance at 750 nm [43].

#### 2.3.5. Oxidative Stress Indices Estimation

The level of gastric malondialdehyde (MDA) as lipid peroxidation indicator was determined in the stomach homogenates guided by the procedure mentioned in Cayman's TBARS assay kit. Protein content of the endogenous antioxidant enzymes, superoxide dismutase (SOD), catalase (CAT), and glutathione peroxidase (GPx), was estimated in the stomach tissue homogenate of all rats following the steps of Cayman colorimetric protocols (Ann Arbor, Michigan, USA).

#### 2.3.6. Prostaglandin E_2_ Level Assay

The level of prostaglandin (PGE_2_) was evaluated in the stomach tissue homogenates according to the instructions detailed in the rat PGE_2_ Elisa immunoassay kit's handbook (Uscn Life Science, Wuhan, China). The assay was conducted using 96-well plate precoated with monoclonal antibody related to rat PGE_2_ antigen. Gastric PGE_2_ level was interpolated from the standard curve obtained at 450 nm in pg/mL, and the protein level of PGE_2_ was calculated for all the results in pg/mg protein.

#### 2.3.7. Stomach Histopathology of Hematoxylin and Eosin

The rats' stomachs fixed in 10% buffered formalin using PBS were further passed through a series of processes in the tissue processing machine followed by microtome-sectioning of the stomachs at 5 *µ*m thickness. One part of the sections was prepared on normal glass slides and stained with hematoxylin and eosin (H&E) to routinely examine gastric lesions [44].

#### 2.3.8. Immunohistochemistry Assessment of COX-2 and iNOS

The other part of sections collected from all animal groups was prepared for immunohistochemistry staining of cyclooxygenase 2 (COX-2) and nitric oxide synthase (iNOS) markers in the stomach tissues. For this purpose, precoated glass slides were heated in microwave oven (Sharp, Bangkok, Thailand) at 60°C for ten minutes. Next, the tissue sections were deparaffinized, rehydrated, and then dipped in sodium citrate buffer microwaved for 10 minutes (10 mM, pH 6.2) for antigen retrieval process. Immunostaining protocol was performed according to the instructions provided by Thermo Fisher Scientific one-in-all kit (Thermo Fisher Scientific, Waltham, Massachusetts, USA). At room temperature, 3% methanolic H_2_O_2_ was applied to the sections and left for 15-minute incubation to block the endogenous peroxidase of the stomach sections. Following peroxidase blocking, the sections were properly washed with double-distilled water and phosphate buffered saline (PBS). The stomach tissue sections were then incubated with rabbit monoclonal antibodies COX-2 (catalog # MA5-14568) and iNOS (catalog # PA1-036) (Thermo Fisher Scientific, Waltham, Massachusetts, USA) and added at dilution rates of 1:100 and 1 : 50, respectively, for 10 minutes in aluminum humid chamber (Thermo Fisher Scientific, Waltham, Massachusetts, USA). After incubation, the tissue sections were carefully and properly washed for 2 minutes with PBST (PBS mixed with 0.05% Tween 20). Diluted DAB chromagen (100 *µ*L) was added to each tissue section, and reincubation repeated for 8 minutes inside the humidified chamber, followed by rinsing the sections with distilled water. For counterstaining, 100 *µ*L of hematoxylin reagent was added to cover each tissue and left for 3-minute incubation inside the humid chamber, followed by distilled-water wash. Finally, the tissue sections were dipped in 0.25% ammonia, rewashed thoroughly with distilled water, and mounted for microscopic examination. Quantitative analysis of the stained antigens was performed using ImageJ software (Adobe Systems Inc., San Jose, CA, USA) according to Jensen, 2013 [45].

#### 2.3.9. Semiquantitative RT-PCR of Gastric TNF-*α* and NF-*κ*B

The expression of TNF-*α* and that of NF-*κ*B in the gastric tissue samples were assessed using semiquantitative real time polymerase chain reaction (qRT-PCR) technique. For this purpose, the RNA samples from the stomach tissues of the seven groups (normal, absolute EtOH, OMP, QP, QG, QB, and QF) were isolated and purified using RNeasy Mini Kit (Qiagen, Texas, USA) and optimized following the instructions of the manual under sterile laboratory conditions. Each tissue sample was disrupted using mortar and pestle tool and homogenized using 0.9 mm sterile syringe needle. The purity of the isolated RNA was determined by uploading 100 *µ*l of each isolated RNA sample into NanoPhotometer P330 (Implen, GmbH, Germany), and the RNA purity was estimated from the ratio between the absorbance readings A260/A280. The purified RNA samples were then reverse-transcribed following the protocol described in SensiFAST cDNA Synthesis Kit (Bioline, Massachusetts, USA) using 1 *µ*g of the isolated RNA per sample. The primers sequenced in Table 1 were utilized in processing qRT-PCR and glyceraldehyde-3-phosphate dehydrogenase (GAPDH) and used as a reference housekeeping gene for normalizing and comparing the resulting values of the target genes. The procedure was performed according to the protocol of SensiFAST SYBR No-ROX Kit (Bioline, Massachusetts, USA). The reaction was prepared by mixing 4 *µ*l of the transcribed cDNA with 10 *µ*l of the provided SensiFAST SYBR No-ROX mix, 4.4 *µ*l distilled water, 0.8 *µ*l forward primer (10 pmol), and 0.8 *µ*l reverse primer (10 pmol) reaching final volume of 20 *µ*l, while the experiment was run in triplicate for convenient analysis. The amplification program of PikoReal PCR machine (Thermo Fisher Scientific, California, USA) was adjusted for 3-step cycles that started at 95°C for 2-minute polymerase activation, followed by 40 cycles at 95°C for 5-second denaturation, 63°C for 10-second annealing, and finally 72°C for 15-second extension. For detection of the resulting gene bands, 1% agarose gel electrophoresis was run, and the intensity of the produced signal was received by gel imaging system (Bio-Rad Gel Doc XR, California, USA). The output results of gene expression were expressed as amplification and melting curves (Supplementary Figures 1(a)–1(b)), while the data were calculated from the cycle threshold (Ct) and presented as fold gene expression using the formulae below [46]:

(2) $$\begin{array}{c} \Delta \text{Ct of sample}=\text{Ct}\left(\text{ target gene}\;\frac{\text{TNF}-\alpha }{\text{Nf}-\kappa \text{B}}\right)-\text{Ct}\left(\text{reference gene GAPDH}\right),\\ \Delta \Delta \text{Ct of sample}=\Delta \text{Ct}\left(\text{treated sample}\right)-\Delta \text{Ct}\left(\text{normal control sample}\right),\\ \text{fold gene expression}=2^{-\left(\Delta \Delta \text{Ct}\right)}. \end{array}$$

### 2.4. Statistical Analysis

Statistically, all the data were analyzed using one-way ANOVA (IBM SPSS Statistics software version 23). Tukey's test analysis was chosen for obtaining the output of results, while all the data were displayed as mean ± SD (*n* = 6). The probability value of *p* ≤ 0.05 was considered significant.

## 3. Results

### 3.1. DPPH Inhibition of the Processed Quinoa Seeds

**Figure 1:**
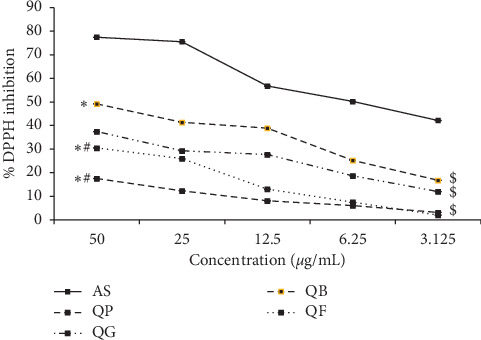
Percent inhibition of the samples, QP (quinoa seeds cooked under high pressure), QG (first stage-germinated quinoa seeds), QB (quinoa seeds fermented by *Lactobacillus plantarum* bacteria), and QF (quinoa seeds fermented by *Rhizopus oligosporus* fungus) to the stable DPPH free radical in comparison to ascorbic acid standard (AS). ^*∗*^*p* < 0.05 compared to AS at the highest concentration (50 *µ*g/mL). ^#^*p* < 0.05 compared to QB at the highest concentration (50 *µ*g/mL). ^$^*p* < 0.05 compared to AS at the lowest concentration (3.125 *µ*g/mL).

The results of % DPPH inhibition of the samples QP, QG, QB, and QF in comparison to the standard AS are displayed in Figure 1. Although the % DPPH inhibition of QP, QG, QB, and QF significantly recorded low values compared to that of the standard AS at all tested concentrations (50–3.125 *µ*g/mL), the values obtained from the different processed seeds remained within the acceptable range. In addition, the highest tested concentration, QB_50_, recorded significantly higher % inhibition value (49.180 ± 0.004 *µ*g/mL) compared to QP_50_ and QF_50_ (17.53 ± 0.023 and 30.517 ± 0.008 *µ*g/mL, respectively), while QG_50_ did not display significance (37.45 ± 0.002 *µ*g/mL) compared to QB_50_. Moreover, at the lowest tested concentration (3.125 *µ*g/mL), all samples (QP_3.125_, QG_3.125_, QB_3.125_, and QF_3.125_) did not show significance compared to each other.

### 3.2. Index of Gastric Ulceration

**Figure 2:**
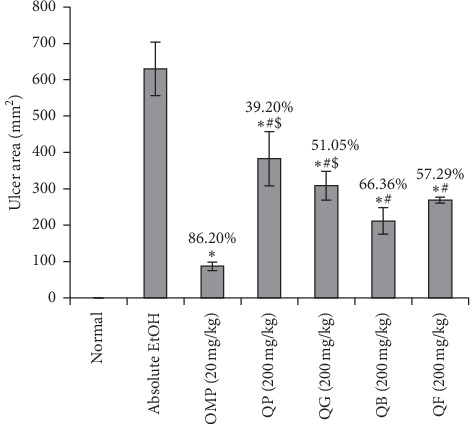
Effect of oral doses of 200 mg/kg of QP (quinoa seeds cooked under high pressure), QG (first stage-germinated quinoa seeds), QB (quinoa seeds fermented by *Lactobacillus plantarum* bacteria), and QF (quinoa seeds fermented by *Rhizopus oligosporus* fungus) on the ulcer index induced by absolute ethanol (absolute EtOH, 5 mL/kg) and in comparison to the reference omeprazole (OMP, 20 mg/kg). ^*∗*^*p* < 0.05 compared to absolute EtOH. ^#^*p* < 0.05 compared to omeprazole OMP. ^$^*p* < 0.05 compared to QB. The percentages of ulcer area inhibition are recorded on the bars.

Generally, the rats of all the groups were alive with variable degrees of health status. Normal group were healthy and active with no abnormal behavioral or toxic symptoms. Absolute EtOH group animals were very weak, while OMP group rats reported good activity. There was clear variation in the health and activity status of the rats given the differently processed quinoa seeds (QP, QG, QB, and QF). QB-treated rats were active and healthy compared to OMP-treated group, while the animals of the other groups showed moderately descending activity from QP and QG to GF. The potential outcomes of the differently processed quinoa seeds (QP, QG, QB, and QF) on the stomach ulcer index are presented in Figure 2. Oral administration of omeprazole at concentration of 20 mg/kg or the samples (QP, QG, QB, and QF) at concentration of 200 mg/kg showed significant inhibition in the severe ulcerated area developed by absolute ethanol. However, reduction of the ulcer index measured from the tested samples (QP, QG, QB, and QF) was significantly lower than that of the reference omeprazole. Additionally, QB recorded insignificant ulcer index compared to QF and significant one compared to QP and QG.

### 3.3. Stomach Macroscopic

**Figure 3:**
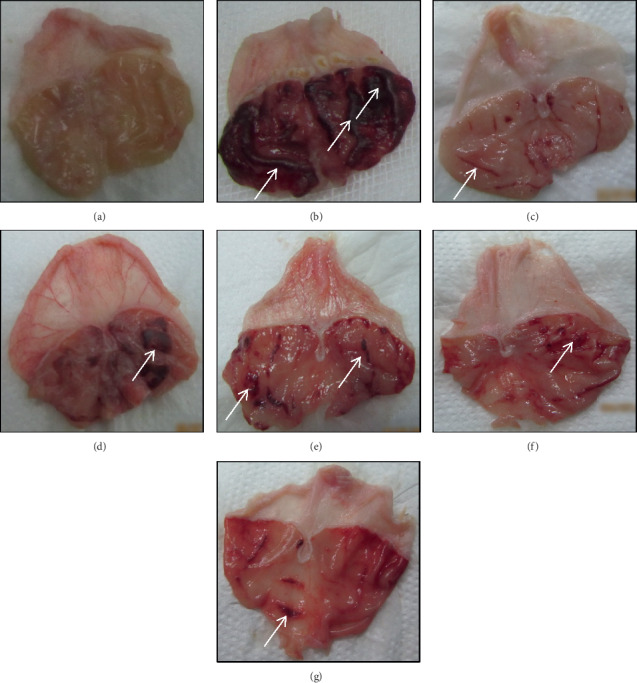
Effect of QP, QG, QB, and QF on the macroscopic status of the rats' stomachs. (a) Normal stomach. (b) Stomach treated with absolute EtOH at dose of 5 mL/kg showing severe haemorrhagic stripped lesions along the ridges of mucosa (white arrow). (c) Stomach treated with omeprazole (20 mg/kg) showing significant reduction in the lesioned strips. (d) Stomach treated with QP (quinoa seeds cooked under high pressure, 200 mg/kg) showing moderated ulcer lesions. (e) Stomach treated with QG (first stage-germinated quinoa seeds, 200 mg/kg) showing mild ulcerated strips. (f) Stomach treated with QB (quinoa seeds fermented by *Lactobacillus plantarum* bacteria, 200 mg/kg) and (g) stomach treated with QF (quinoa seeds fermented by *Rhizopus oligosporus* fungus, 200 mg/kg) showing clear reduction in lesion areas of the stomachs.

The macroscopic photographs of the rats' stomachs resulting from oral administration of QP, QG, QB, and QF (200 mg/kg) are illustrated in Figure 3. The stomach from absolute EtOH group showed obviously serious haemorrhagic lesions arranged as strips along the ridges of mucosa indicating severe ulceration. Pretreatment of the rats with QP, QG, QB, and QF resulted in considerable and variable reduction in the gastric lesion areas, while QB-treated stomach showed comparable mucosal protection compared to OMP-treated stomach.

### 3.4. Histopathology of H&E

**Figure 4:**
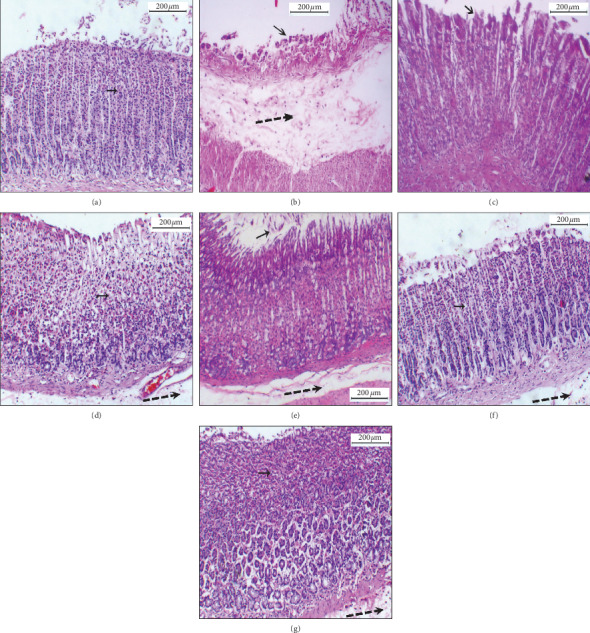
Effect of QP, QG, QB, and QF on the microscopic hematoxylin and eosin (H&E) staining of stomach sections. (a) Normal stomach. (b) Stomach treated with absolute EtOH at dose of 5 mL/kg showing intense ulceration and degeneration of upper gastric mucosa (small straight arrow) along with necrotic gastric glands (double-lined arrow) and swollen edematous submucosa (dashed arrow). (c) Stomach treated with omeprazole (20 mg/kg) showing significant reduction in the damage of the upper mucosa (straight arrow) and maintained gastric glands. (d) Stomach treated with QP (quinoa seeds cooked under high pressure, 200 mg/kg) showing moderated ulceration and upper mucosal degeneration. (e) Stomach treated with QG (first stage-germinated quinoa seeds, 200 mg/kg) showing mild necrosis of gastric glands. (f) Stomach treated with QB (quinoa seeds fermented by *Lactobacillus plantarum* bacteria, 200 mg/kg) showing minor damage of the gastric mucosa. (g) Stomach treated with QF (quinoa seeds fermented by *Rhizopus oligosporus* fungus, 200 mg/kg) showing mild mucosal damage.

The results of staining with hematoxylin and eosin of the stomachs collected from all groups of animals at the end of the experiment are displayed in Figure 4. Absolute EtOH at dose of 5 mL/kg caused aggressive damage and degeneration of the gastric mucosal part with marked edematous submucosa. Oral administration of omeprazole (20 mg/kg) or the different types of processed quinoa seeds (QP, QG, QB, and QF) at dose of 200 mg/kg has obviously inhibited the mucosal damage via reduction of degenerated upper mucosal part, necrotic gastric glands, and submucosal edematous area. QB showed the most gastroprotective potential followed by QF and QG, while QP revealed the least gastroprotective potential. However, the submucosal edematous area remained comparable between the stomach sections from all types of processed quinoa (QP, QG, QB, and QF).

### 3.5. Gastric pH and GWM

**Figure 5:**
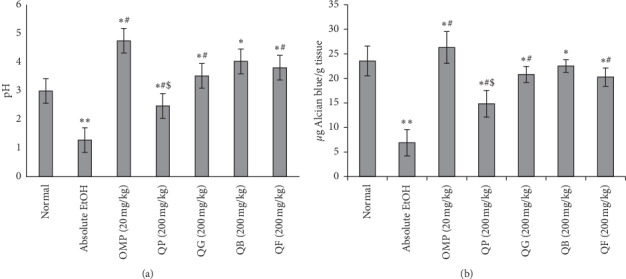
Effect of oral doses of 200 mg/kg of QP (quinoa seeds cooked under high pressure), QG (first stage-germinated quinoa seeds), QB (quinoa seeds fermented by *Lactobacillus plantarum* bacteria), and QF (quinoa seeds fermented by *Rhizopus oligosporus* fungus) on the (a) gastric pH and (b) gastric wall mucus (GWM) in absolute EtOH-induced (5 mL/kg) acute gastric injury in rats and in comparison with the reference drug omeprazole (OMP, 20 mg/kg). ^*∗∗*^*p* < 0.05 compared to normal.^*∗*^*p* < 0.05 compared to absolute EtOH. ^#^*p* < 0.05 compared to omeprazole OMP. ^$^*p* < 0.05 compared to QB.

The responses of gastric pH and gastric wall mucus measured from the stomachs of experimental rats upon administration of QP, QG, QB, and QF compared to the insult absolute EtOH and the reference drug OMP are charted in Figures 5(a) and 5(b). The outcomes revealed that treating the rats' stomachs with OMP or the processed seeds (QP, QG, QB, and QF) has remarkably altered the harmful effect of absolute EtOH on pH (Figure 3(a)) and GWM (Figure 3(b)). In comparison with OMP (pH = 4.74 and GWM = 26.29 ± 3.24 *µ*g Alcian blue/g tissue), QP, QG, and QF showed significantly lower pH (2.47 ± 0.40, 3.52 ± 6.21, and 3.80 ± 0.56, respectively) and GWM (14.82 ± 2.72, 20.80 ± 1.63, and 20.26 ± 1.86 *µ*g Alcian blue/g tissue, respectively). On the other hand, QB treatment revealed insignificance in the values obtained from estimating both parameters (pH = 4.02 ± 0.53 and GWM = 22.53 ± 1.29 *µ*g Alcian blue/g tissue) compared to OMP. Furthermore, the pH and GWM data measured from QB were significantly higher than those from QP without significance recorded in comparison with QG and QF.

### 3.6. Gastric MDA, Antioxidant Enzymes, and PGE_2_

Potency of the different types of processed quinoa seeds (QP, QG, QB, and QF) on the protein level of MDA, endogenous antioxidant enzymes (SOD, CAT, and GPx), and PGE_2_ is tabulated in Table 2. The presented data showed that absolute ethanol injuriously and remarkably increased the protein level of MDA and decreased the level of PGE_2_ as well as the activity of gastric antioxidant enzymes. Oral administration of OMP and the processed quinoa seeds (QP, QG, QB, and QF) has significantly reversed the protein content of MDA and CAT. Though the protein content of SOD, GPx, and PGE_2_ in the rats fed with QP group recorded higher values than that of absolute EtOH group, no significance was detected between both groups on analysis of the resulting data. In comparison with the measured parameters from OMP group, MDA level from QP, QG, and QF rats was significantly higher, while SOD activity of QP and QG groups, CAT activity of QP and QF, and PGE_2_ level of QP group were significantly low. The protein content of the studied parameters from QB group displayed insignificance when compared to OMP group. Additionally, no significance was reported in the GPx values between QG, QB, and QF groups. Further, QP group documented higher MDA value and lower SOD and PGE_2_ values compared to QB group.

### 3.7. Immunohistochemistry of Stomach COX-2 and iNOS

**Figure 6:**
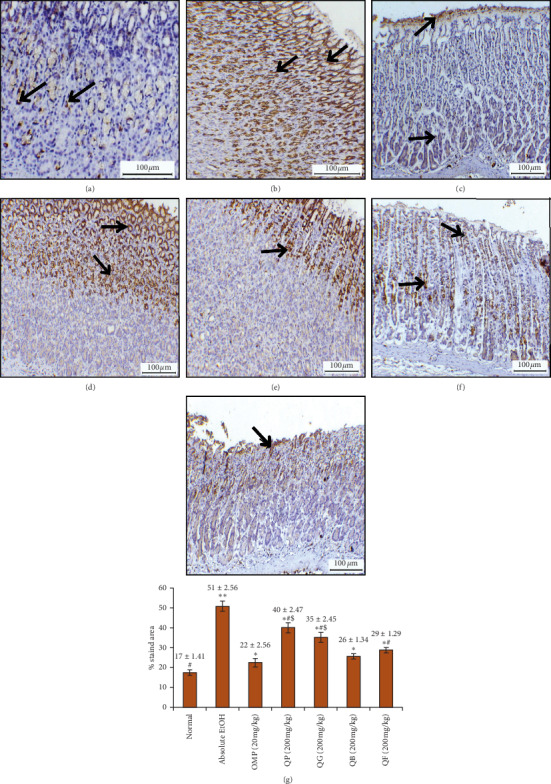
Effect of QP, QG, QB, and QF on the immunostaining of cyclooxygenase 2 (COX-2) enzyme in stomach sections from all animal groups. (a) Slight expression of COX-2 in normal stomach. (b) Marked overexpression of COX-2 in the gastric mucosa with intensive brown staining towards the upper part of stomach treated with absolute EtOH at dose of 5 mL/kg (black arrow). (c) Slightly expressed COX-2 in the lower part of the gastric mucosa with fine brown-stained layer and COX-2 expression on the mucosal surface from the stomach treated with omeprazole (20 mg/kg). (d) Moderately immunoexpressed COX-2 in the upper half of the mucosal part from the stomach treated with QP (quinoa seeds cooked under high pressure, 200 mg/kg). (e) Stomach treated with QG (first stage-germinated quinoa seeds, 200 mg/kg) showing mild expression of COX-2 in the top quarter of the gastric mucosa. (f) Stomach treated with QB (quinoa seeds fermented by *Lactobacillus plantarum* bacteria, 200 mg/kg) showing considerable reduction in immuno-COX-2 expression in the upper part compared to the lower part of the gastric mucosa. (g) Stomach treated with QF (quinoa seeds fermented by *Rhizopus oligosporus* fungus, 200 mg/kg) showing more expression of COX-2 in the upper part of the gastric mucosa than the lower part. Quantitative estimation of immunohistochemical staining based on the determination of the % positive-stained area analyzed from 6 images/group using ImageJ analysis software is charted down the images. ^*∗∗*^*p* < 0.05 compared to normal. ^*∗*^*p* < 0.05 compared to absolute EtOH. ^#^*p* < 0.05 compared to omeprazole OMP. ^$^*p* < 0.05 compared to QB.

**Figure 7:**
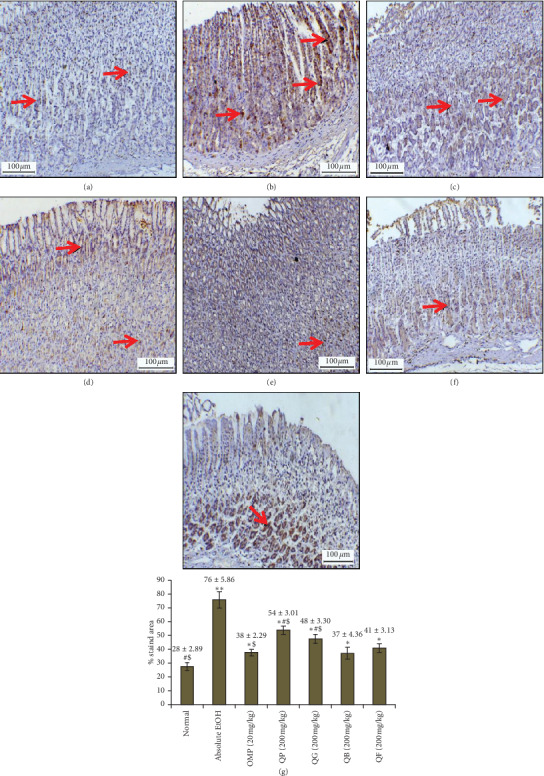
Effect of QP, QG, QB, and QF on the immunostaining of nitric oxide synthase (iNOS) enzyme in stomach sections from all animal groups. (a) Slight expression of iNOS in normal stomach. (b) Marked overexpression of iNOS throughout the whole mucosal part of the stomach treated with absolute EtOH at dose of 5 mL/kg (red arrow). (c) Slightly expressed iNOS in the stomach treated with omeprazole (20 mg/kg). (d) Moderately immunoexpressed iNOS in the stomach treated with QP (quinoa seeds cooked under high pressure, 200 mg/kg). (e) Stomach treated with QG (first stage-germinated quinoa seeds, 200 mg/kg) showing mild expression of iNOS in the gastric mucosa. (f) Stomach treated with QB (quinoa seeds fermented by *Lactobacillus plantarum* bacteria, 200 mg/kg) showing more immuno-iNOS expression in the lower part than the upper part of gastric mucosa. (g) Stomach treated with QF (quinoa seeds fermented by *Rhizopus oligosporus* fungus, 200 mg/kg) showing clear expression of iNOS in the upper part with minor expression in the lower part of the gastric mucosa. Quantitative estimation of immunohistochemical staining based on the determination of the % positive-stained area from 6 images/group using ImageJ analysis software is charted down the images. ^*∗∗*^*p* < 0.05 compared to normal. ^*∗*^*p* < 0.05 compared to absolute EtOH. ^#^*p* < 0.05 compared to omeprazole OMP. ^$^*p* < 0.05 compared to QB.

Results of the impact of pretreating the rats with omeprazole (OMP) or the four types of processed quinoa seeds (QP, QG, QB, QF) on the immune-expression of COX-2 and iNOS markers are illustrated in Figures 6 and 7, respectively. Immunostaining of COX-2 showed slightly expressed COX-2 in the normal stomach and intensive staining and aggressive overexpression of COX-2 in the stomach from absolute EtOH group. The immunostained stomachs from OMP or QP, QG, QB, QF displayed marked reduction in the COX-2 expression. Additionally, immunostained tissue sections from QB did not show significance compared to those from OMP group as quantitatively analyzed in Figure 6. Similarly, iNOS enzyme revealed minor expression in normal gastric mucosa and intensive expression in the stomach tissues from absolute EtOH group (Figure 7). On the other hand, overexpression of the same protein was significantly reduced in the stomachs pretreated with OMP or any of the processed quinoa seeds (QP, QG, QB, QF), indicative of inhibited inflammation as confirmed by the quantitative analysis of % stained area in Figure 7. Stomach tissue samples from QB and QF showed insignificant iNOS staining compared to OMP group.

### 3.8. RT-PCR of TNF-*α* and NF-*κ*B

**Figure 8:**
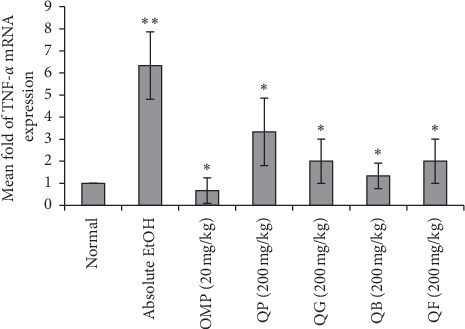
Effect of the four types of processed quinoa seeds on the expression of TNF-*α* gene in the stomach tissues of experimental rats. Fold change of TNF-*α* gene expression normalized to GAPDH as a reference housekeeping gene. ^*∗∗*^*p* < 0.05 compared to normal. ^*∗*^*p* < 0.05 compared to absolute EtOH.

**Figure 9:**
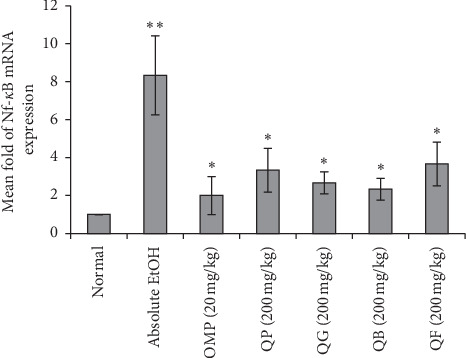
Effect of the four types of processed quinoa seeds on the expression of NF-*κ*B gene in the stomach tissues of experimental rats. Fold change of NF-*κ*B gene expression normalized to GAPDH as a reference housekeeping gene. ^*∗∗*^*p* < 0.05 compared to normal. ^*∗*^*p* < 0.05 compared to absolute EtOH.

The effect of pretreatment of the rats with the different types of processed quinoa seeds on gene expression results of TNF-*α* and NF-*κ*B in their gastric mucosal cells after ethanol-induced ulceration is shown in Figures 8 and 9, respectively. Based on the results, TNF-*α* and NF-*κ*B were highly expressed in the stomach tissues of absolute EtOH group reaching 6 ± 1.53 and 8 ± 2.08-fold, respectively, compared to normal stomach. Oral administration of OMP or the processed quinoa seeds (QP, QG, QB, QF) has obviously and significantly downexpressed the target genes without showing significance between the treated groups or in comparison to normal stomach.

## 4. Discussion

In the past few decades, Scientists focused on the benefits of edible seeds and cereals for human health [47]. At the epidemiology level, studies showed that routine intake of grains and their products can protect against many chronic ailments such as cancer, type 2 diabetes, and heart diseases [48, 49] via their contents of antioxidants and phytochemicals [47]. Additionally, researchers reported that whole grains and/or their isolated compounds exhibit gastroprotective activity against different ulcerogenic insults [22, 50–52].

Quinoa seeds are characterized by higher nutritional components compared to other traditional cereals [13]. Additionally, they contain high percentage of bioactive peptides that showed marked antiradical power and antioxidant activity in *in vitro* studies [28]. Previous studies reported that raw quinoa seeds are rich in phenolic compounds and betanins with increasing concentration in colored seeds compared to white strains [19]. Processing techniques of quinoa seeds can considerably improve their antioxidant activity [25]. Researchers showed that germination of seeds refined the antioxidant capacity of unprocessed quinoa seeds compared to yeast-fermentation process [26]. However, the current study revealed that *Lactobacillus* fermentation (QB) was significantly effective in increasing the % DPPH inhibition compared to germination (QG) and *Rhizopus* fermentation (QF). These results may be attributed to the proteolysis process performed by *Lactobacillus* bacteria during fermentation of quinoa seeds and the release of high proportion of their antioxidant amino acid constituents [28]. Further, the significant incompatibility between the DPPH scavenging power of QB and QF may refer to the variable effect of the selected microorganism used in fermentation process on the release of more phenolic compounds from the *Lactobacillus*-fermented seeds than *Rhizopus*-fermented ones [26]. Although pressure-cooking plays a role in upgrading the antioxidant efficacy of quinoa seeds as previously reported [25], it was significantly low in the present study in comparison with germination and fermentation processing as indicated by the lowest % DPPH value. The resulting DPPH scavenging activity of quinoa seeds in this study may refer to the high scavenging activity of their constituents of flavonols and their glycosides [15], ecdysteroids [53], and betanins [14].

In many preclinical studies, absolute ethanol induced serious lesions in the gastric mucosa at the oral dose administered (5 mL/kg) [54, 55]. In this study, the same dose of absolute EtOH promoted severe ulceration as indicated by the highest ulcer index and lowest pH and GWM. These parameters were significantly altered by oral pretreatment of the rats with QP, QG, QB, and QF at dose of 200 mg/kg suggesting marked gastroprotection. The significant gastroprotective potentiality of QB and QF on ulcer index and pH compared to QG and QP can refer to the elevated percentages of antioxidant peptides and phytochemicals released from the fermented seeds and detected by the higher % DPPH scavenging power in comparison with pressure-cooking and germination processes as previously published [25, 28]. The augmentative ability of processed quinoa seeds (QP, QG, QB, and QF) to produce GWM may refer to the enhancement activity of their polysaccharide constituents through increased secretion of mucus and/or formation of defensive covering on the mucosal surface, which is consistent with the findings of Cordiero et al. [22].

Oxidative stress plays key role in the pathogenesis of gastric mucosal damage developed by ethanol via direct formation of free radicals, attenuation of endogenous antioxidant enzymes of stomach cells, and elevation of lipid peroxidation of cell membranes leading to necrosis and cell death [56]. In the present study, the pathogenic effect of absolute EtOH on the stomach of rats was translated into considerable reduction in the gastric content of SOD, CAT, and GPx along with remarkable increase in MDA level. The reversed results obtained from oral administration of QP, QG, QB, and QF to the rats, close to omeprazole-treated group, may be accredited to the enhancing effect of the different processing techniques in releasing the antioxidant ingredients from the processed seeds [26] and sufficiently boosting the enzymatic secretion of endogenous antioxidant enzymes to inhibit oxidative stress [57]. Additionally, studies showed that red quinoa seeds contain about 15 *µ*g/g total carotenoids and 55 *µ*g/g total vitamin E which are plenty enough to exhibit antioxidant and cytoprotective activities against necrotizing agents [19, 51]. Moreover, the protective effect of QP, QG, QB, and QF on the gastric mucosal architecture was confirmed by the moderately to mildly observed macroscopic lesions and histopathology aberrations of the treated rats' stomachs.

Protective prostaglandins as chief derivatives of cyclooxygenase isoforms (COX-1 and COX-2) are cytoprotective mediators *in vivo* and *in vitro*, playing crucial role in maintaining the health of the stomach through stimulation of mucus and bicarbonate production, enhancement of stomach microcirculation, and attenuation of gastric mucosal injury [58, 59]. Recent studies confirmed this notion of prostaglandin-mediated gastroprotection against ethanol-induced gastric injury [56, 60, 61]. The notable improvement in the protein level of gastric PGE_2_ that accompanied the pretreatment of QP, QG, QB, and QF in the current study can be regarded as the variable release of vitamin E (tocopherols) from the processed red quinoa seeds and its magnifying effect on the secretion of PGE_2_ from stomach cells [62, 63].

Inflammation is one of the factors that cause damage to the body through cascade of inflammatory mediators such as TNF-*α*, COX-2, and iNOS [64]. TNF-*α* is considered one of the aggressive inflammatory and injurious cytokines in stomach damage induced by absolute EtOH [65]. In addition to alleviation of blood flow around the ulcer area and intensifying inflammation, TNF-*α* activates NF-*κ*B that incorporates in the signal transduction inflammatory pathway activating other inflammatory genes, including COX-2 and iNOS, and amplifying ulceration [66–68]. According to Chen et al. and Pandit, COX-2 plays a role in gastric mucosal inflammation through the release of inflammatory prostaglandins [69, 70]. COX-2 expeditiously responds to ulcerative factors such as nonsteroidal anti-inflammatory drugs (NSAIDSs and ethanol). Upon stimulation, COX-2 is expressed rapidly in the gastric mucosa and increasingly within short period of time resulting in severe inflammation [64]. Increased formation of iNOS in the ulcer area is sufficient enough to increase the production of reactive oxygen species (ROS) and potentiate injury [71]. In the current study, absolute EtOH overexpressed the level of the inflammatory indices TNF-*α*, NF-*κ*B, COX-2, and iNOS in the gastric tissues of rats. Pretreatment of rats with OMP or QP, QG, QB, QF significantly inhibited the overexpression of these inflammatory parameters and attenuated inflammatory responses. The inhibitory activity of the different types of processed red quinoa seeds close to omeprazole efficacy can be referred to the gastroprotective and anti-inflammatory activities of flavonoids, phenolic acids [64, 66, 67], and carotenoids [72, 73] released variably from the processed red seeds [8, 9] via blocking the signaling pathway of TNF-*α* and inhibition of the inflammatory mediators involved (COX-2 and iNOS) [74]. These findings are consistent with the results of previous studies [75, 76] on the anti-inflammatory effect of the phenolic compounds of quinoa seeds through the inhibitory effect of inflammatory mediators TNF-*α*, NF-*κ*B, iNOS, and COX-2. In addition, saponin contents of quinoa seeds may be the reason of the inhibitory activity of the inflammatory mediators tested in the present study and revealed by other studies [77].

## 5. Conclusions

Taken together, the outcomes of the present study revealed that the differently processed quinoa seeds exhibited acceptable DPPH scavenging capacity with regard to their constituents of flavonoids, polyphenols, and carotenoids. Additionally, the seeds showed gastroprotective potential against absolute ethanol-induced acute gastric injury via reduction of ulcer index; increase of the gastric pH, gastric wall mucus secretion, PGE_2_, and endogenous antioxidant enzymes (SOD, CAT, and GPx); inhibition of lipid peroxidation level; and expression of inflammatory mediators (TNF-*α*, NF-*κ*B, COX-2, and iNOS). Effect of the different processing methods on the gastroprotective activity of quinoa seeds in this research revealed that the protective activity of QB (quinoa seeds fermented by *Lactobacillus plantarum* bacteria) against gastric injury was the highest activity, followed by QF (quinoa seeds fermented by *Rhizopus oligosporus* fungus) and QG (first stage-germinated quinoa seeds), while QP (quinoa seeds cooked under high pressure) revealed the lowest activity. Further mechanistic studies are required to explain the variation in the gastroprotective potential of the differently processed quinoa seeds.

## Figures and Tables

**Table 1:** Oligonucleotide primer sequences used in conducting qRT-PCR of TNF-*α* and NF-kB gene expression protocol.

Gene	Forward primer (5′-3′)	Reverse primer (5′-3′)	Product number	^*b*^ *T*_*a*_	bp
GAPDH	TTGTGCAGTGCCAGCCTCGT	TGCCGTTGAACTTGCCGTGG	NM_017008.4	60	201
TNF-*α*	TCTTCAAGGGACAAGGCTGC	CTTGATGGCAGAGAGGAGGC	NM_012675.3	60	104
NF-*κ*B	CCCAAGTACCCGGATACAGC	GGGCAACTCATCTTCCGTGA	NM_001105720.2	60	124

GAPDH: glyceraldehyde-3-phosphate dehydrogenase (housekeeping gene), TNF-*α*: tumor necrosis factor alpha, NF-*κ*B: nuclear factor kappa enhancer of activated B cells, ^*b*^*T*_*a*_: annealing temperature, bp: gene length in base pairs.

**Table 2:** Effect of oral administration of the four types of processed quinoa seeds (QP, QG, QB, and QF) on the gastric protein content of MDA, SOD, CAT, GPx, and PGE_2_.

Treatment	MDA	SOD	CAT	GPx	PGE_2_
	(nmol/mg protein)	(U/mg protein)	(nmol/min/mg protein)	(nmol/min/mg protein)	(pg/mg protein)
Normal	30.06 ± 6.01	21.42 ± 5.89	57.64 ± 11.65	945.14 ± 213.04	553.41 ± 133.27
Absolute EtOH	135.42 ± 19.08^*∗∗*^	9.64 ± 1.02^*∗∗*^	15.08 ± 5.31^*∗∗*^	481.02 ± 115.87^*∗∗*^	187.66 ± 62.48^*∗∗*^
OMP (20 mg/kg)	30.56 ± 5.48^*∗*^	22.83 ± 2.77^*∗*^	67.57 ± 15.44^*∗*^	962.66 ± 174.33^*∗*^	566.12 ± 106.14^*∗*^
QP (200 mg/kg)	96.24 ± 13.12^*∗*#$^	12.30 ± 5.39^#$^	46.96 ± 7.45^*∗*#^	765.38 ± 188.28	310.85 ± 22.37^#$^
QG (200 mg/kg)	71.56 ± 42.95^*∗*#^	13.70 ± 5.42^#^	54.47 ± 6.79^*∗*^	958.88 ± 158.87^*∗*^	477.59 ± 79.37^*∗*^
QB (200 mg/kg)	46.86 ± 15.64^*∗*^	20.95 ± 4.94^*∗*^	59.78 ± 5.84^*∗*^	969.72 ± 145.37^*∗*^	592.19 ± 99.37^*∗*^
QF (200 mg/kg)	69.62 ± 14.02^*∗*#^	18.03 ± 3.20^*∗*^	37.63 ± 8.68^*∗*#$^	874.73 ± 132.38^*∗*^	498.57 ± 69.19^*∗*^

^*∗∗*^ *p* < 0.05 compared to normal. ^*∗*^*p* < 0.05 compared to absolute EtOH. ^#^*p* < 0.05 compared to omeprazole OMP. ^$^*p* < 0.05 compared to QB. QP (quinoa seeds cooked under high pressure); QG (first stage-germinated quinoa seeds); QB (quinoa seeds fermented by *Lactobacillus plantarum* bacteria); QF (quinoa seeds fermented by *Rhizopus oligosporus* fungus).

## Data Availability

The data used to support the findings of this study are available from the corresponding author upon request.

## Abbreviations

ANOVA: Analysis of variance
BSA: Bovine serum albumen standard
CAT: Catalase
cDNA: Complementary deoxyribonucleic acid
COX-1: Cyclooxygenase 1
COX-2: Cyclooxygenase 2
Ct: Cycle threshold
DAB: 3,3′-Diaminobenzidine
DMSO: Dimethyl sulphoxide
DPPH: 2,2-Diphenyl-1-picrylhydrazyl
EtOH: Absolute ethanol
FAO: Food and Agriculture Organization
GAPDH: Glyceraldehyde-3-phosphate dehydrogenase
GPx: Glutathione peroxidase
GWM: Gastric wall mucus
H&E: Hematoxylin and eosin
IAO: International Accreditation Organization
iNOS: Nitric oxide synthase
NCER: Nile Center of Experimental Research
NF-*κ*B: Nuclear factor kappa of B cells
NSAIDSs: Nonsteroidal anti-inflammatory drugs
OMP: Omeprazole
PBS: Phosphate buffered saline
PBST: Phosphate buffered saline mixed with 0.05% Tween 20
PGE_2_: Prostaglandin E_2_
QB: *Lactobacillus plantarum* bacteria-fermented quinoa seeds
QF: *Rhizopus oligosporus* fungus-fermented quinoa seeds
QG: First stage-germinated quinoa seeds
QP: Pressure-cooked quinoa seeds
qRT-PCR: Semiquantitative real time polymerase chain reaction
RNA: Ribonucleic acid
RT-PCR: Reverse transcription polymerase chain reaction
SD: Sprague Dawley
SOD: Superoxide dismutase
TBARS: Thiobarbituric acid reactive substances
TNF-*α*: Tumor necrosis factor alpha.

## Supplementary Materials

Supplementary Materials Figure 1: (A) amplification curve showing the cycle number at which the PCR fluorescence signal is generated corresponding to doubling of TNF-*α* gene in the samples; (B) melting curve showing that the change in fluorescence signal is generated corresponding to the melting of TNF-*α* gene with respect to temperature change; (C) amplification curve showing the cycle number at which the PCR fluorescence signal is generated corresponding to doubling of NF-*κ*B gene in the samples; (D) melting curve showing that the change in fluorescence signal is generated corresponding to the melting of NF-*κ*B gene with respect to temperature change. Figure 2: quantitative analysis of hematoxylin and eosin (H&E) staining of the stomach tissues. OMP (omeprazole, 20 mg/kg); QP (quinoa seeds cooked under high pressure, 200 mg/kg); QG (first stage-germinated quinoa seeds, 200 mg/kg); QB (quinoa seeds fermented by *Lactobacillus plantarum* bacteria, 200 mg/kg); QF (quinoa seeds fermented by *Rhizopus oligosporus* fungus, 200 mg/kg). Results were illustrated based on the determination of the % positive-stained area analyzed from 6 images/group using ImageJ analysis software. ^*∗∗*^*p* < 0.05 compared to normal. ^*∗*^*p* < 0.05 compared to absolute EtOH. ^#^*p* < 0.05 compared to omeprazole OMP. ^$^*p* < 0.05 compared to QB.
