# Supplementary material for: The Efficacy of Processing Strategies on the Gastroprotective Potentiality of Chenopodium quinoa Seeds
Source: ScientificWorldJournal. 2020 May 28;2020:6326452. doi: 10.1155/2020/6326452 (PMC7275209; doi:10.1155/2020/6326452)
Supplement: Supplementary Materials — Figure 1: (A) amplification curve showing the cycle number at which the PCR fluorescence signal is generated corresponding to doubling of TNF-α gene in the samples; (B) melting curve showing that the change in fluorescence signal is generated corresponding to the melting of TNF-α gene with respect to temperature change; (C) amplification curve showing the cycle number at which the PCR fluorescence signal is generated corresponding to doubling of NF-κB gene in the samples; (D) melting curve showing that the change in fluorescence signal is generated corresponding to the melting of NF-κB gene with respect to temperature change. Figure 2: quantitative analysis of hematoxylin and eosin (H&E) staining of the stomach tissues. OMP (omeprazole, 20 mg/kg); QP (quinoa seeds cooked under high pressure, 200 mg/kg); QG (first stage-germinated quinoa seeds, 200 mg/kg); QB (quinoa seeds fermented by Lactobacillus plantarum bacteria, 200 mg/kg); QF (quinoa seeds fermented by Rhizopus oligosporus fungus, 200 mg/kg). Results were illustrated based on the determination of the % positive-stained area analyzed from 6 images/group using ImageJ analysis software. ∗∗p < 0.05 compared to normal. ∗p < 0.05 compared to absolute EtOH. #p < 0.05 compared to omeprazole OMP. $p < 0.05 compared to QB. [file 6326452.f1.pdf]

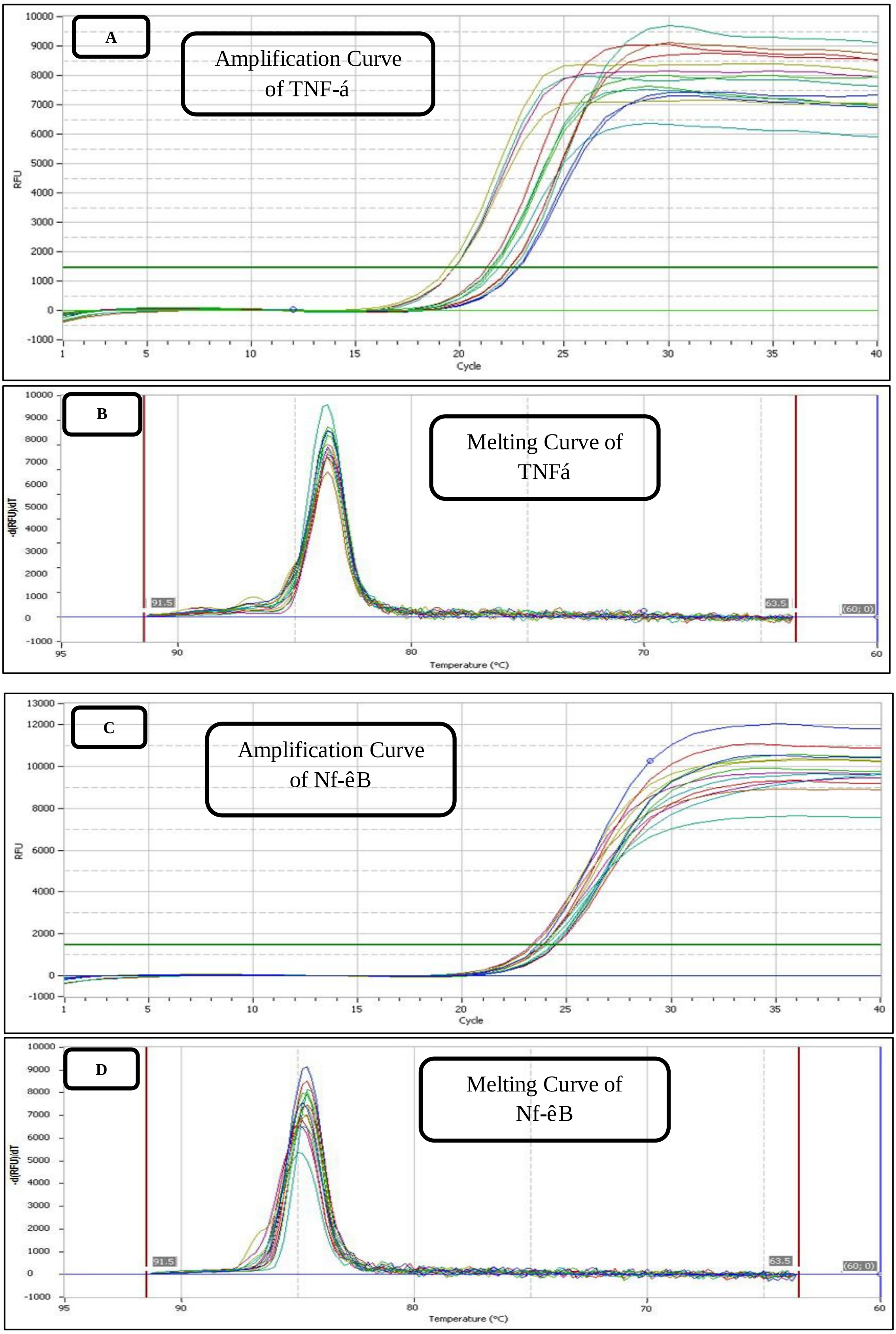

Create PDF files with PDF Writer for Windows 8. This is an evaluation copy. Buy full version now.

Figure 1: (A) Amplification curve showing the cycle number at which the PCR fluorescence signal is generated in correspondence to doubling of TNF-á gene in the samples. (B) Melting curve showing the change in fluorescence signal is generated in correspondence to the melting of TNF-á gene with respect to temperature change.(C) Amplification curve showing the cycle number at which the PCR fluorescence signal is generated in correspondenceto doubling of Nf-êBgene in the samples. (D) Melting curve showing the change in fluorescence signal is generated in correspondence to the melting of Nf-êBgene with respect to temperature change.

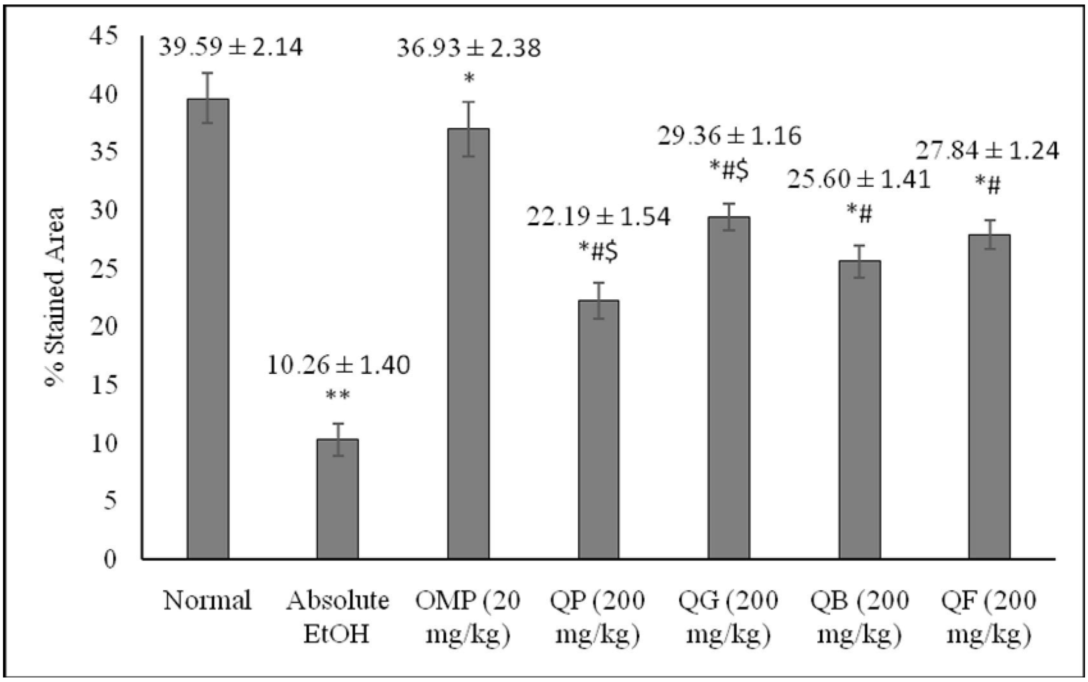

Figure 2: Quantitative analysis of haematoxylin and eosin (H & E) staining of the stomach tissues. OMP (Omeprazole, 20 mg/kg); QP (quinoa seeds cooked under high pressure, 200 mg/kg);QG (first stage-germinated quinoa seeds, 200 mg/kg); QB (quinoa seeds fermented by *Lactobacillusplantarum* bacteria, 200 mg/kg); QF (quinoa seeds fermented by *Rhizopusoligosporus* fungus, 200 mg/kg). Results were illustratedbased on the determination of the % positive-stained area analyzed from 6 images/group using Image-J analysis software. **P <0.05 compared to normal. *P <0.05 compared to Absolute EtOH. $^{\#}$P <0.05 compared to omeprazole OMP. $^{\$}$P <0.05 compared to QB.

Create PDF files with PDF Writer for Windows 8. This is an evaluation copy. Buy full version now.
